# Supplementary material for: The E3 ligase Riplet promotes RIG-I signaling independent of RIG-I oligomerization
Source: Nat Commun. 2023 Nov 11;14:7308. doi: 10.1038/s41467-023-42982-0 (PMC10640585; doi:10.1038/s41467-023-42982-0)
Supplement: Supplementary file 1 — Supplementary Information [file 41467_2023_42982_MOESM1_ESM.pdf]

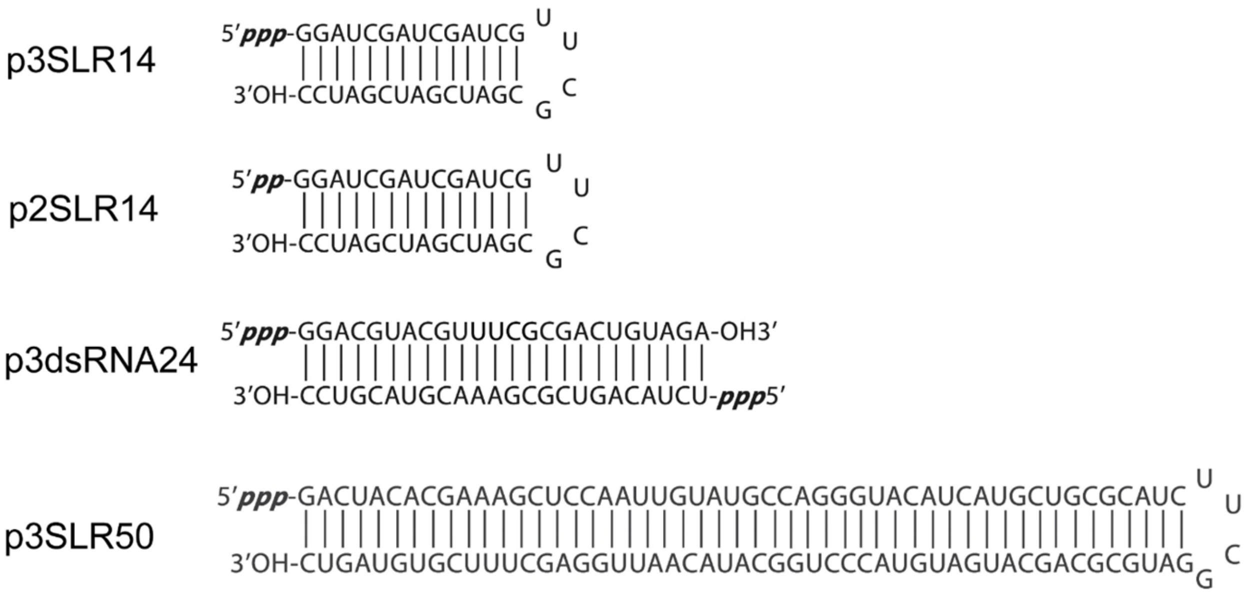

Supplementary Fig. 1: RNA duplexes used in this study. Sequence of RNA duplexes used in this study.

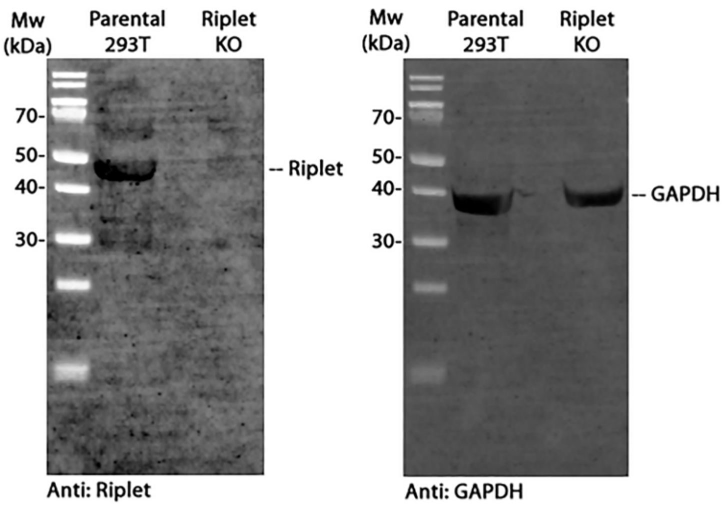

Supplementary Fig. 2: Validation of Riplet-KO cell line. Immunoblotting of Riplet in both parental and Riplet-KO HEK293T cells. This experiment was independently repeated once.

a

	RIG-I:p3SLR14			RIG-I:p3dsRNA24			Apo-RIG-I		
	K_a ($M^{-1}s^{-1}$)	K_d (s^{-1})	K_D (uM)	K_a ($M^{-1}s^{-1}$)	K_d (s^{-1})	K_D (uM)	K_a ($M^{-1}s^{-1}$)	K_d (s^{-1})	K_D (uM)
Riplet-FL	1.9×10^4	0.013	0.7	4.5×10^5	0.003	6.6 nM	4.6×10^3	0.0034	0.7
ΔRING	5.4×10^4	0.067	1.2	8.1×10^5	0.0085	10 nM	1.1×10^5	0.073	0.7
PrySpry	5.1×10^4	0.18	3.6	1.3×10^5	0.19	1.4	6.0×10^4	0.18	3.1

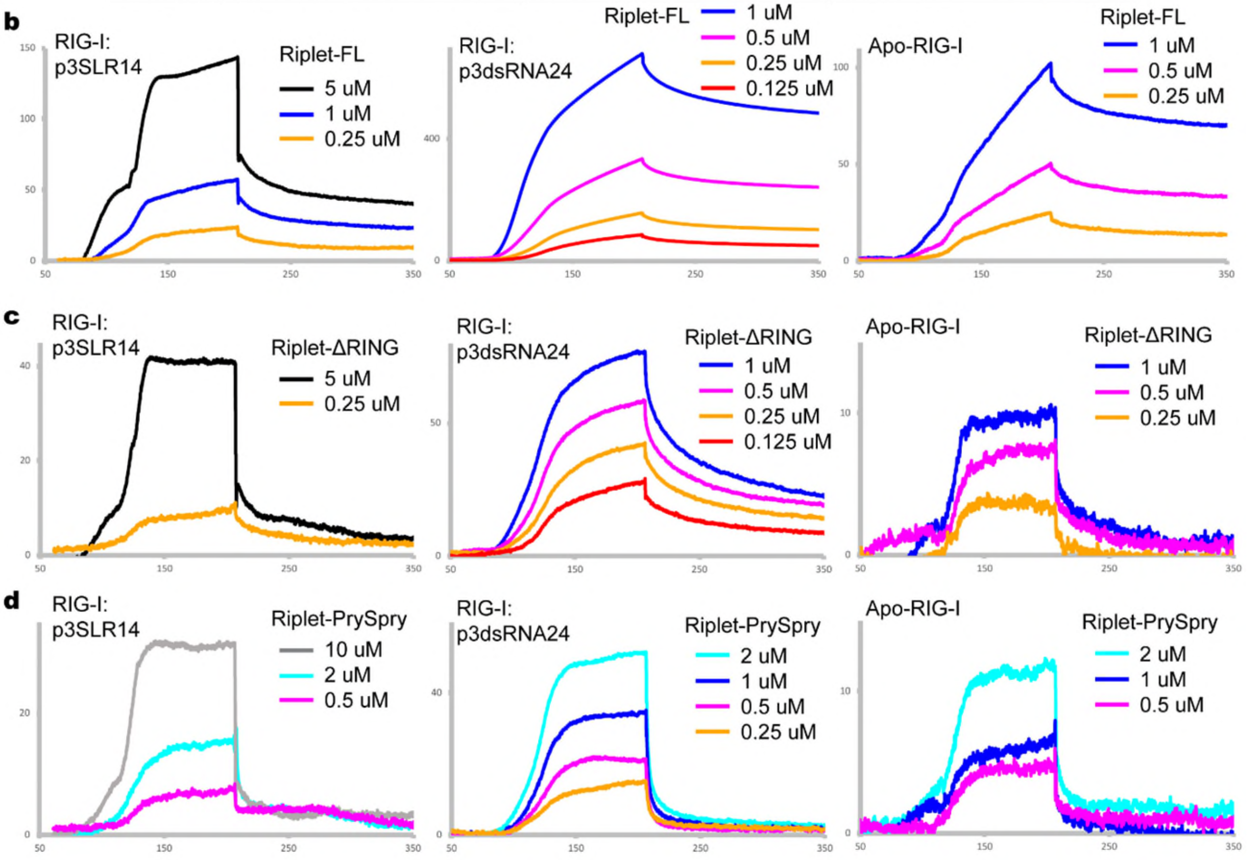

Supplementary Fig. 3: Measurement of Riplet binding to RIG-I. a Kinetics of Riplet binding to RIG-I:p3SLR14, RIG-I:p3dsRNA24, and apo-RIG-I. **b-d** Binding curves of Riplet-FL (**b**), Riplet-ΔRING (**c**), and Riplet-PrySpry (**d**) to RIG-I:p3SLR14, RIG-I:p3dsRNA24, and apo-RIG-I

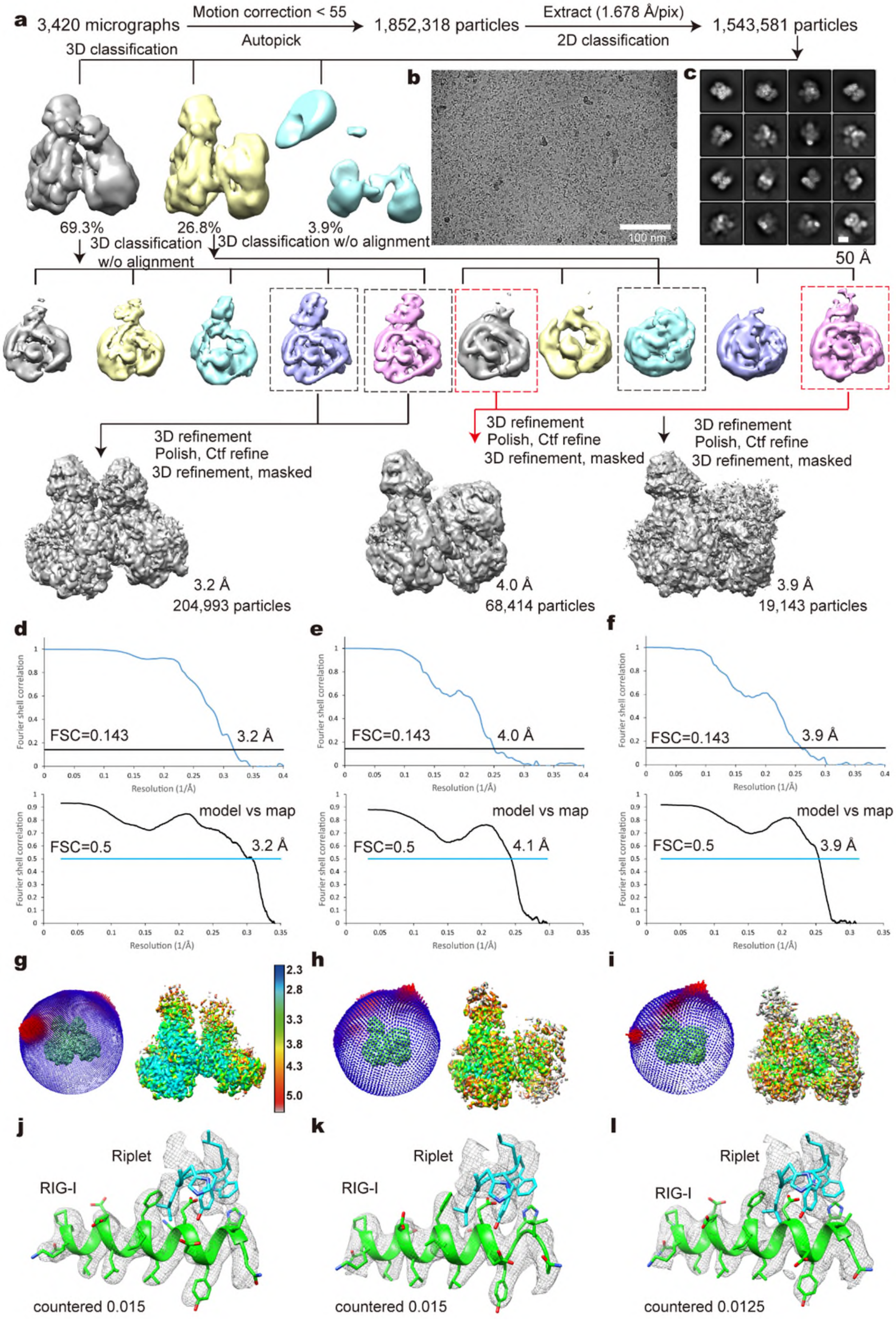
a
3,420 micrographs
Motion correction < 55
Autopick
1,852,318 particles
Extract (1.678 Å/pix)
2D classification
1,543,581 particles
3D classification
69.3%
26.8%
3.9%
3D classification w/o alignment
3D classification w/o alignment
b
100 nm
c
50 Å
3D refinement
Polish, Ctf refine
3D refinement, masked
3.2 Å
204,993 particles
4.0 Å
68,414 particles
3.9 Å
19,143 particles
d
e
f
Fourier shell correlation
Resolution (1/Å)
FSC=0.143
3.2 Å
4.0 Å
3.9 Å
model vs map
FSC=0.5
3.2 Å
4.1 Å
3.9 Å
g
h
i
2.3
2.8
3.3
3.8
4.3
5.0
j
k
l
Riplet
RIG-I
countered 0.015
countered 0.015
countered 0.0125

Supplementary Fig. 4: Cryo-EM analysis of the RIG-I:p3dsRNA24:Riplet complex a Flow chart of cryo-EM data processing. **b** Representative micrograph. **c** Representative 2D class averages from Relion 2D classification. **d-f** Fourier shell correlation (FSC) curve and fourier shell correlation between map and model for the end-end complex (**d**), end-transition complex (**e**) and end-inter complex (**f**). **g-i** Euler angle distribution of all particles used in the final map reconstruction for the end-end complex (**g**), end-transition complex (**h**) and end-inter complex (**i**). The local resolution display of each complex is illustrated. **j-l** Representative local densities of the end-end complex (**j**), the end-transition complex (**k**) and the end-inter complex (**l**).

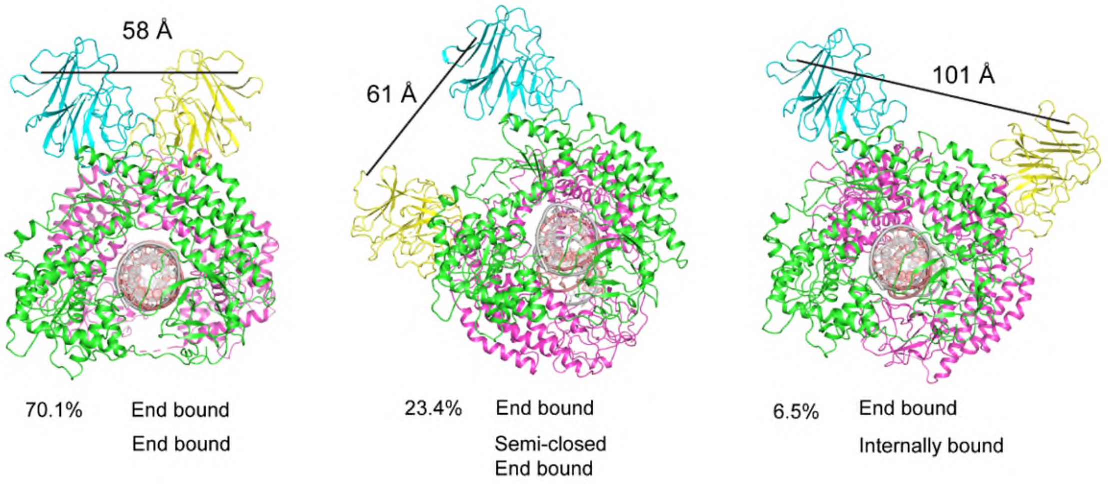

Supplementary Fig. 5: Riplet CC domain accommodates a range of distances between two PrySpry domains. The distances between the N-terminal of two PrySpry domains in three structures are denoted.

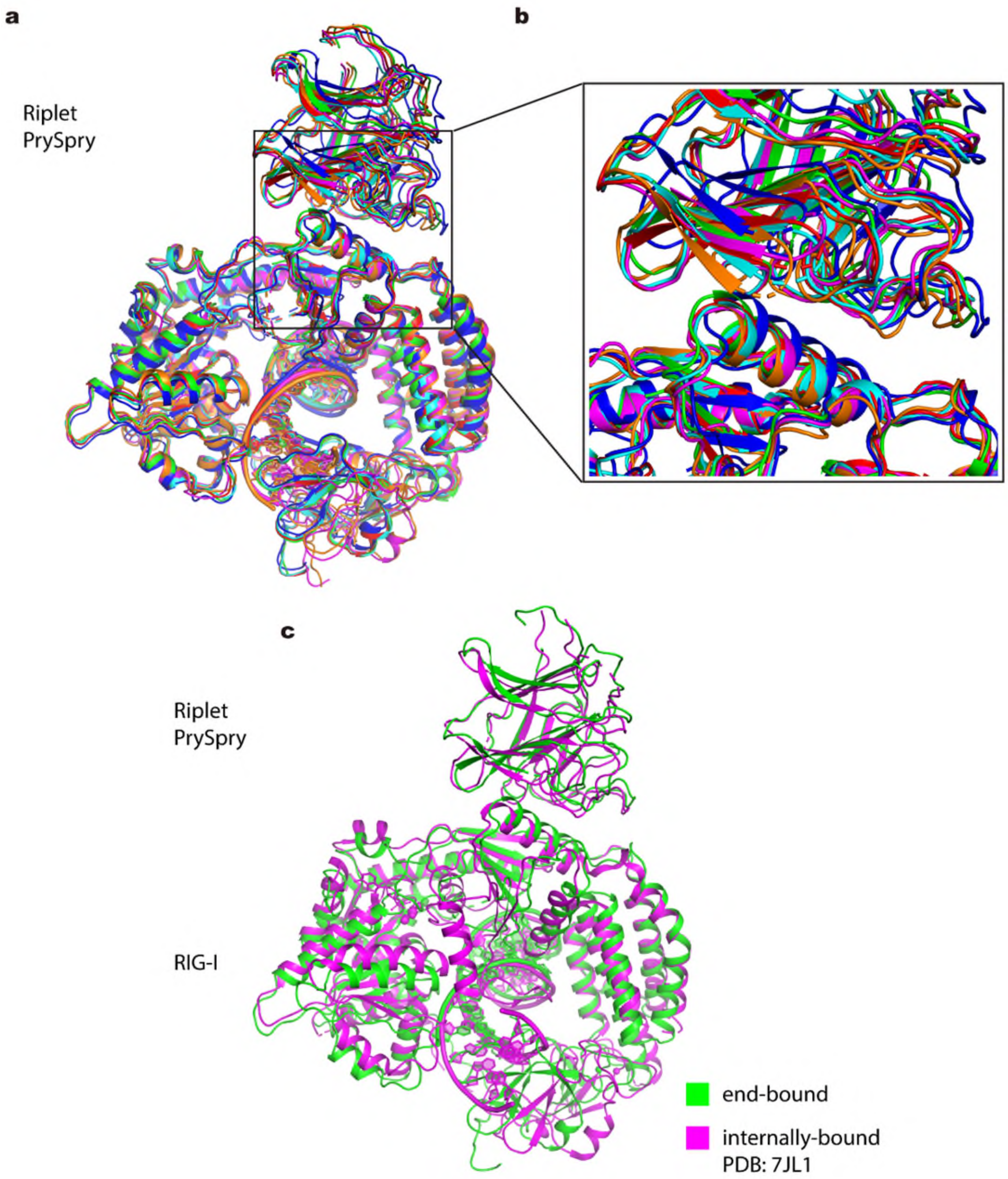

Supplementary Fig. 6: Riplet PrySpry recognizes RIG-I Hel2 similarly regardless of RIG-I binding sites. a Overlay of halves from three RIG-I:p3dsRNA24:Riplet structures. Half 1 and Half 2 of end-end, end-transition, end-inter complexes are superimposed and rendered in red and green, blue and magenta, cyan and orange, respectively. **b** Zoom-in views of the RIG-I:Riplet interface in the overlaid structures. **c** Overlay of half from end-end RIG-I:p3dsRNA24:Riplet complex and previously published internally-bound RIG-I:RNA:Riplet complex.

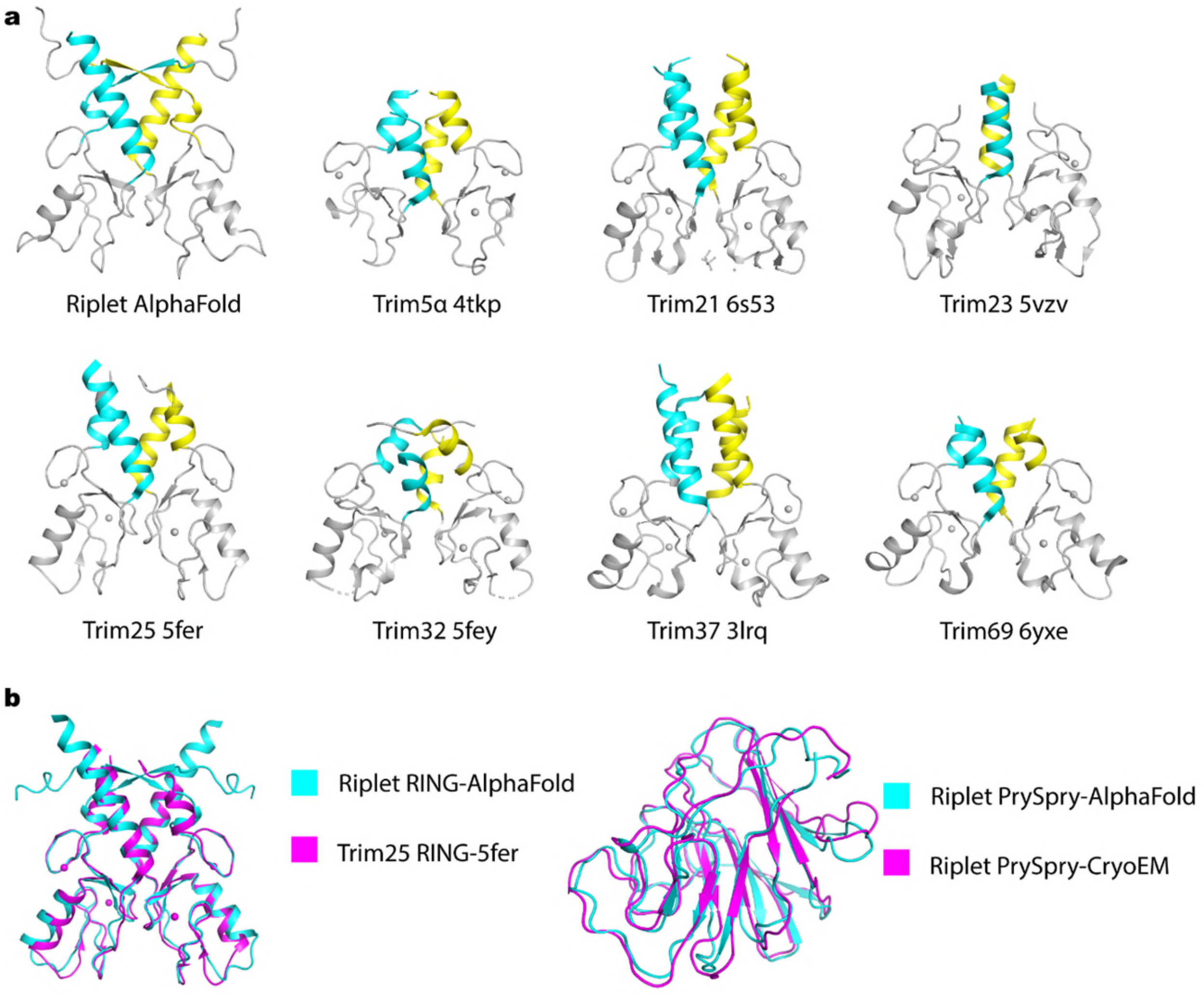

Supplementary Fig. 7: Comparison of predicted and resolved structures. a Overview of RING dimer from predicted Riplet structure and determined structures. The N-terminal, C-terminal helices and β-sheet are colored. **b** Overlay of predicted and resolved structures. The predicted Riplet RING and PrySpry are superimposed to Trim25 RING and resolved Riplet PrySpry structures, respectively.

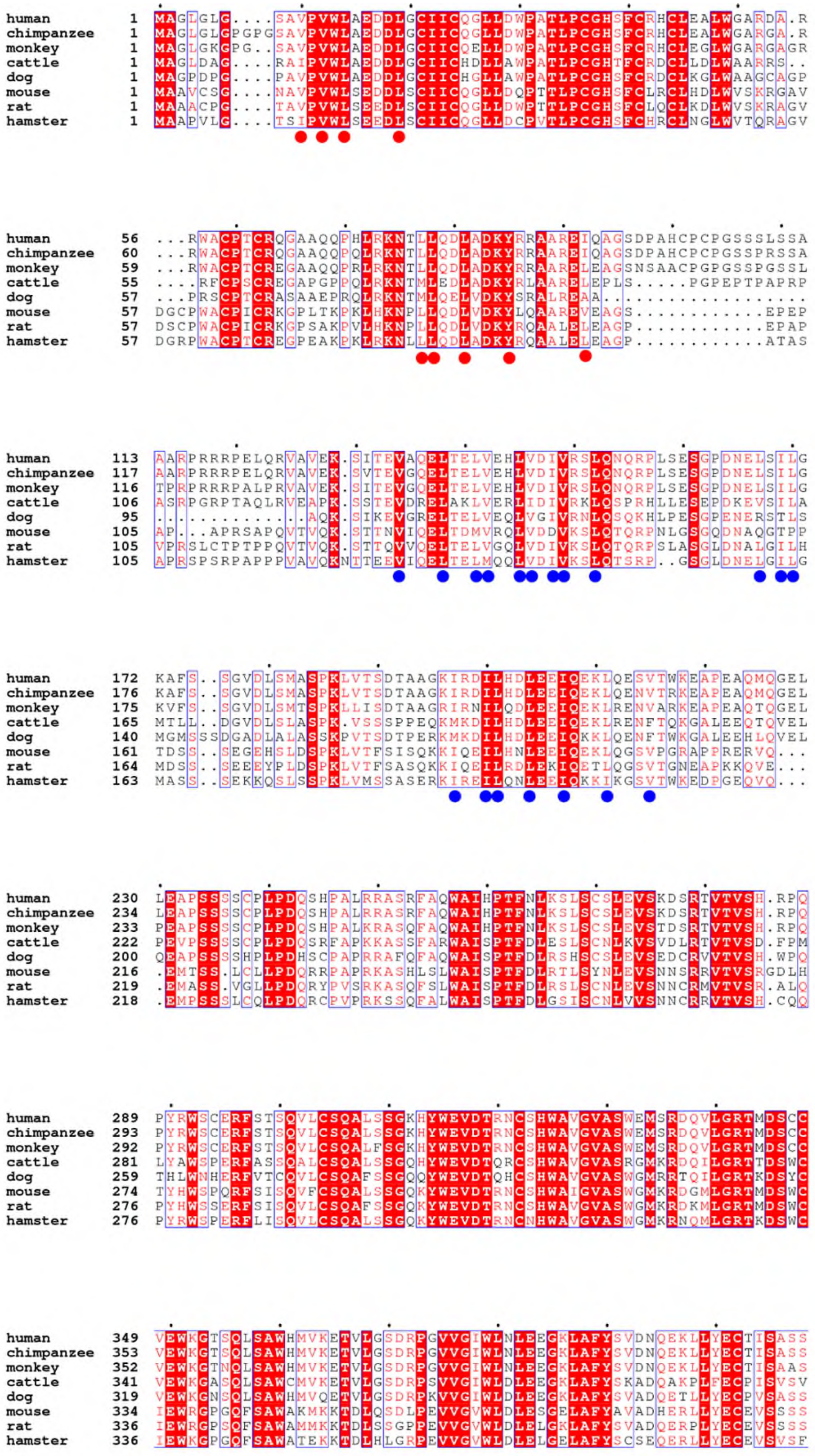

human 1 MAGLGLG....SAVPVWLAEDDLGCIICQGLLDWPATLPCGHSFCRHCLEALWGARDA.R
chimpanzee 1 MAGLGLGPGPGSAVPVWLAEDDLGCIICQGLLDWPATLPCGHSFCRHCLEALWGARGA.R
monkey 1 MAGLGKGPG..SAVPVWLAEDDLGCIICQELLDWPATLPCGHSFCRHCLEGLWGARGAGR
cattle 1 MAGLDAG....RAIPVWLAEDDLGCIICHDLLAWPATLPCGHTFCRDCLLDLWAARRS..
dog 1 MAGPDPG....PAVPVWLAEDDLGCIICHGLLAWPATLPCGHSFCRDCLKGLWAAGCAGP
mouse 1 MAAVCSG....NAVPVWLSEDDLSCIICQGLLDQPTTLPCGHSFCLRCLHDLWVSKRGAV
rat 1 MAAACPG....TAVPVWLSEEDLSCIICQGLLDWPTTLPCGHSFCLQCLKDLWVSKRAGV
hamster 1 MAAPVLG....TSIPVWLSEEDLSCIICQGLLDCPVTLPCGHSFCHRCLNGLWVTQRAGV
human 56 ...RWACPTCRQGAAQQPHLRKNTLLQDLADKYRRAAREIQAGSDPAHCPCPGSSSLSSA
chimpanzee 60 ...RWACPTCRQGAAQQPQLRKNTLLQDLADKYRRAAREIQAGSDPAHCPCPGSSPRSSA
monkey 59 ...RWACPTCREGAAQQPRLRKNTLLQDLADKYRRAARELEAGSNSAACPGPGSSPGSSL
cattle 55 ....RFCPSCREGAPGPPQLRKNTMLEDLADKYRLAARELEPLS.....PGPEPTPAPRP
dog 57 ...PRSCPTCRASAAEPRQLRKNTMLQELVDKYSRALREAA..................
mouse 57 DGCPWACPICRKGPLTKPKLHKNPLLQDLVDKYLQAAREVEAGS............EPEP
rat 57 DSCPWACPICRKGPSAKPVLHKNPLLQDLVDKYRQAALELEAGP............EPAP
hamster 57 DGRPWACPTCREGPEAKPKLRKNLLLQDLADKYRQAALELEAGP............ATAS
human 113 AARPRRRPELQRVAVEK.SITEVAQELTELVEHLVDIVRSLQNQRPLSESGPDNELSILG
chimpanzee 117 AARPRRRPELQRVAVEK.SVTEVGQELTELVEHLVDIVRSLQNQRPLSESGPDNELSILG
monkey 116 TPRPRRRPALPRVAVEK.SITEVGQELTELVEHLVDIVRSLQNQRPLSESGPDNELSILG
cattle 106 ASRPGRPTAQLRVEAPK.SSTEVDRELAKLVERLIDIVRKLQSPRHLLESEPDKEVSILA
dog 95 ....AQK.SIKEVGRELTELVEQLVGIVRNLQSQKHLPESGPENERSTLS
mouse 105 AP...APRSAPQVTVQK.STTNVIQELTDMVRQLVDDVKSLQTQRPNLGSGQDNAQGTPP
rat 105 VPRSLCTPTPPQVTVQK.STTQVVQELTELVGQLVDIVKSLQTQRPSLASGLDNALGILH
hamster 105 APRSPSRPAPPPVAVQKNTTEEVIQELTELMQQLVDIVKSLQTSRP..GSGLDNELGILG
human 172 KAFS..SGVDLSMASPKLVTSDTAAGKIRDILHDLEEIQEKLQESVTWKEAPEAQMQGEL
chimpanzee 176 KAFS..SGVDLSMASPKLVTSDTAAGKIRDILHDLEEIQEKLQENVTRKEAPEAQMQGEL
monkey 175 KVFS..SGVDLSMTSPKLLISDTAAGRIRNILQDLEEIQEKLRENVARKEAPEAQTQGEL
cattle 165 MTLL..DGVDLSLASPK.VSSSPPEQKMKDILHDLEEIQEKLRENFTQKGALEEQTQVEL
dog 140 MGMSSSDGADLALASSKPVTSDTPERKMKDILHDLEEIQKKLQENFTWKGALEEHLQVEL
mouse 161 TDSS..SEGEHSLDSPKLVTFSISQKKIQEILHNLEEIQEKLQGSVPGRAPPRERVQ...
rat 164 MDSS..SEEEYPLDSPKLVTFSASQKKIQEILRDLEKIQETLQGSVTGNEAPKKQVE...
hamster 163 MASS..SEKKQSLSSPKLVMSSASERKIREILQNLEEIQKKIKGSVTWKEDPGEQVQ...
human 230 LEAPSSSSCPLPDQSHPALRRASRFAQWAIHPTFNLKSLSCSLEVSKDSRTVTVSH.RPQ
chimpanzee 234 LEAPSSSSCPLPDQSHPALRRASRFAQWAIHPTFNLKSLSCSLEVSKDSRTVTVSH.RPQ
monkey 233 PEAPSSSSCPLPDQSHPALKRASQFAQWAIHPTFNLKSLSCSLEVSTDSRTVTVSH.RPQ
cattle 222 PEVPSSSSCPLPDQSRFAPKKASSFARWAISPTFDLESLSCNLKVSVDLRTVTVSD.FPM
dog 200 QEAPSSSSHPLPDHSCPAPRRAFQFAQWAISPTFDLRSHSCSLEVSEDCRVVTVSH.WPQ
mouse 216 .EMTSS.LCLLPDQRRPAPRKASHLSLWAISPTFDLRTLSYNLEVSNNSRRVTVSRGDLH
rat 219 .EMASS.VGLLPDQRYPVSRKASQFSLWAISPTFDLRSLSCNLEVSNNCRMVTVSR.ALQ
hamster 218 .EMPSSSLCQLPDQRCPVPRKSSQFALWAISPTFDLGSISCNLVVSNNCRRVTVSH.CQQ
human 289 PYRWSCERFSTSQVLCSQALSSGKHYWEVDTRNCSHWAVGVASWEMSRDQVLGRTMDSCC
chimpanzee 293 PYRWSCERFSTSQVLCSQALSSGKHYWEVDTRNCSHWAVGVASWEMSRDQVLGRTMDSCC
monkey 292 PYRWSCERFSTSQVLCSQALFSGKHYWEVDTRNCSHWAVGVASWEMSRDQVLGRTMDSCC
cattle 281 LYAWSPERFASSQALCSQALSSGQHYWEVDTQRCSHWAVGVASRGMKRDQILGRTTDSWC
dog 259 THLWNHERFVTCQVLCSQAFSSGQQYWEVDTQHCSHWAVGVASWGMRRTQILGRTKDSYC
mouse 274 TYHWSPQRFSISQVFCSQALSSGQKYWEVDTRNCSHWAIGVASWGMKRDGMLGRTMDSWC
rat 276 PYHWSSERFSISQVLCSQAFSSGQKYWEVDTRNCSHWAVGVASWGMKRDKMLGRTMDSWC
hamster 276 PYRWSPERFLISQVLCSQALSSGQKYWEVDTRNCNHWAVGVASWGMKRNQMLGRTKDSWC
human 349 VEWKGTSQLSAWHMVKETVLGSDRPGVVGIWLNLEEGKLAFYSVDNQEKLLYECTISASS
chimpanzee 353 VEWKGTSQLSAWHMVKETVLGSDRPGVVGIWLNLEEGKLAFYSVDNQEKLLYECTISASS
monkey 352 VEWKGTNQLSAWHMVKETVLGSDRPGVVGIWLNLEEGKLAFYSVDNQEKLLYECTISAAS
cattle 341 VEWKGASQLSAWCMVKETVLGSDRPSVVGIWLDLEEGKLAFYSKADQAKPLFECPISVSV
dog 319 VEWKGNSQLSAWHMVQETVLGSDRPKVVGIWLDLEEGKLAFYSVADQETLLYECPVSASS
mouse 334 IEWRGPGQFSAWAKMKKTDLQSDLPEVVGVWLDLESGELAFYAVADHERLLYECEVSSSS
rat 336 IEWRGPSQFSAWAMMKKTDLSSGPPEVVGVWLDLELGKLAFYSVADQERPLYECEVSSSS
hamster 336 IEWKGPGQFSAWATEKKTDLHLGRPEVVGVWLDLELGELAFYSCSEQERLLYECEVSVSF

Supplementary Fig. 8: Sequence alignment of Riplet among mammals. The conserved hydrophobic residues located in the dimeric interface of RING and CC domains are denoted with red and blue spheres, respectively.

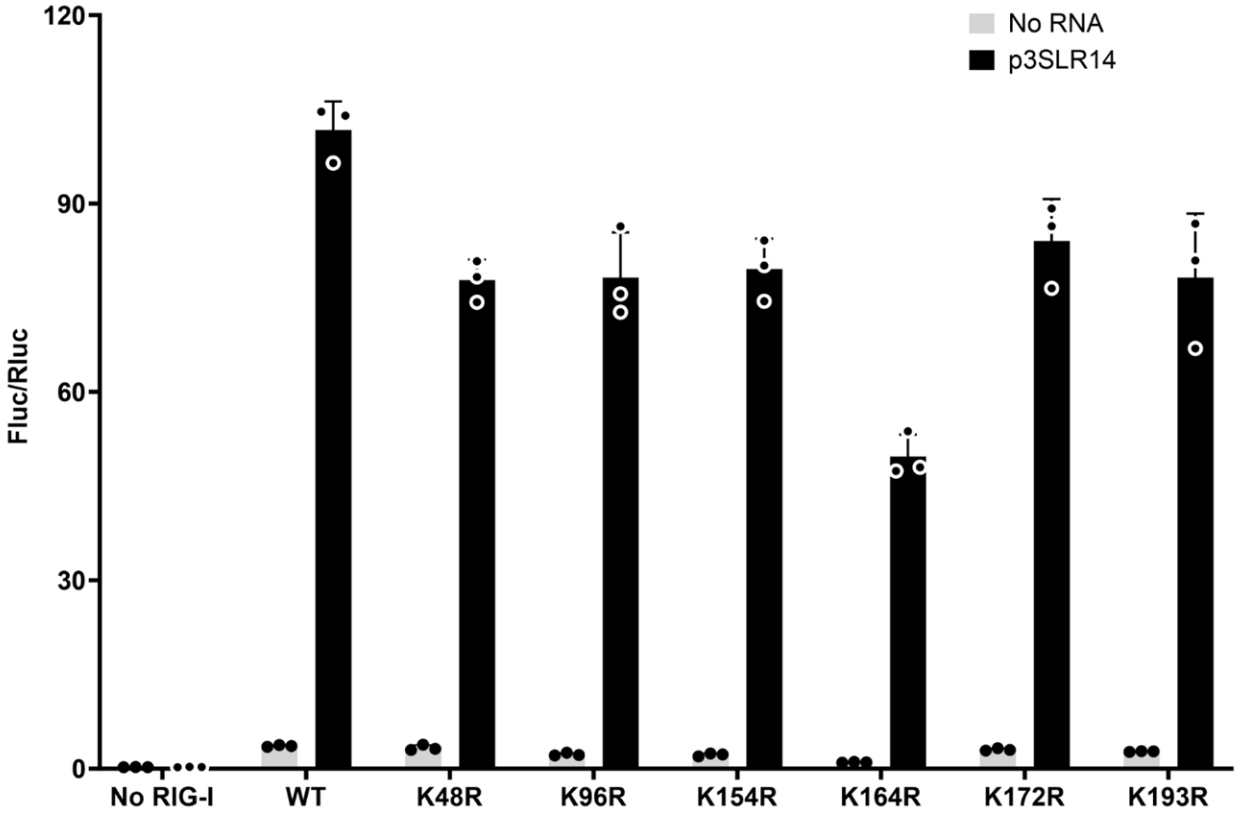

Supplementary Fig. 9: Effect of single point mutants of ubiquitination sites on IFN activation. IFN-β induction by transfection of RIG-I mutants (Lys, ubiquitination sites) stimulated with p3SLR14, OHSLR14 and p3dsRNA24 in parental HEK293T Cells. Data are represented as mean ± SD (n=3 replicates).

Supplementary Table 1. RNA sequences used in this study.

RNAs	Sequences
p3SLR14	5'ppp-GGAUCGAUCGAUCGUUCGCGAUCGAUCGAUCC-OH3'
p2SLR14	5'pp-GGAUCGAUCGAUCGUUCGCGAUCGAUCGAUCC-OH3'
p3SLR50	5'ppp-GACUACACGAAAGCUCCAAUUGUAUGCCAGGGUACAUCAUGCUGCGCAUCUUCGGAUGCGCAGCAUGAUGUACCCUGGCAUACAAUUGGAGCUUUCGUGUAGUC-OH3'
p3dsRNA24a	5'ppp-GGACGUACGUUUCGCGACUGUAGA-OH3'
p3dsRNA24b	5'ppp-UCUACAGUCGCGAAACGUACGUCC-OH3'

Supplementary Table 2. Kinetics of Riplet binding to RIG-I.

	RIG-I:p3SLR14			RIG-I:p3dsRNA24			Apo-RIG-I		
	K_a ($M^{-1}s^{-1}$)	K_d (s^{-1})	K_D (uM)	K_a ($M^{-1}s^{-1}$)	K_d (s^{-1})	K_D (uM)	K_a ($M^{-1}s^{-1}$)	K_d (s^{-1})	K_D (uM)
Riplet-FL	1.9×10^4	0.013	0.7	4.5×10^5	0.003	6.6 nM	4.6×10^3	0.0034	0.7
ΔRING	5.4×10^4	0.067	1.2	8.1×10^5	0.0085	10 nM	1.1×10^5	0.073	0.7
Pspry	5.1×10^4	0.18	3.6	1.3×10^5	0.19	1.4	6.0×10^4	0.18	3.1

Supplementary Table 3. Cryo-EM data collection, refinement and validation statistics.

	End_end (EMDB-29823) (PDB 8G7T)	End_semi-closed-end (EMDB-29824) (PDB 8G7U)	End_inter (EMDB-29825) (PDB 8G7V)
**Data collection and processing**			
Magnification		105,000 ×	
Voltage (kV)		300	
Electron exposure (e–/Å^2)		60	
Defocus range (μm)		-1.0 ~ -2.5	
Pixel size (Å)		0.839	
Symmetry imposed	C1	C1	C1
Initial particle images (no.)		1,852,320	
Final particle images (no.)	204,993	68,414	19,143
Map resolution (Å)	3.2	4.0	3.9
FSC threshold	0.143	0.143	0.143
Map resolution range (Å)	2.3-5.0	2.3-5.0	2.3-5.0
**Refinement**			
Initial model used (PDB code)	7TNX, 7JL1	8G7T	8G7T, 7TO2
Model resolution (Å)	3.1/3.2	3.9/4.1	3.7/3.9
FSC threshold	0.143/0.5	0.143/0.5	0.143/0.5
Model resolution range (Å)			
Map sharpening *B* factor (Å^2)	-76.000	-131.389	-88.730
Model composition			
Non-hydrogen atoms	14059	14229	14007
Protein residues	1619	1634	1621
RNA residues	48	48	46
Ligands	2	2	2
B factors (Å^2)			
Protein	81.60	69.72	73.37
RNA	54.62	58.02	53.46
Ligand	40.25	82.88	59.02
R.m.s. deviations			
Bond lengths (Å)	0.003	0.007	0.006
Bond angles (°)	0.564	0.934	0.834
Validation			
MolProbity score	1.69	1.92	1.72
Clashscore	4.29	8.60	5.87
Poor rotamers (%)	0.60	0.10	0.00
Ramachandran plot			
Favored (%)	96.80	92.80	93.98
Allowed (%)	3.20	7.20	6.02
Disallowed (%)	0.00	0.00	0.00
