# Supplementary material for: The E3 ligase Riplet promotes RIG-I signaling independent of RIG-I oligomerization
Source: Nat Commun. 2023 Nov 11;14:7308. doi: 10.1038/s41467-023-42982-0 (PMC10640585; doi:10.1038/s41467-023-42982-0)
Supplement: Supplementary file 3 — Reporting Summary [file 41467_2023_42982_MOESM3_ESM.pdf]

nature portfolio

Corresponding author(s): Anna Marie Pyle

Last updated by author(s): Oct 9, 2023

Reporting Summary

Nature Portfolio wishes to improve the reproducibility of the work that we publish. This form provides structure for consistency and transparency in reporting. For further information on Nature Portfolio policies, see our Editorial Policies and the Editorial Policy Checklist.

Statistics

For all statistical analyses, confirm that the following items are present in the figure legend, table legend, main text, or Methods section.

n/a	Confirmed	
☐	☒	The exact sample size (n) for each experimental group/condition, given as a discrete number and unit of measurement
☐	☒	A statement on whether measurements were taken from distinct samples or whether the same sample was measured repeatedly
☒	☐	The statistical test(s) used AND whether they are one- or two-sided<br>*Only common tests should be described solely by name; describe more complex techniques in the Methods section.*
☒	☐	A description of all covariates tested
☒	☐	A description of any assumptions or corrections, such as tests of normality and adjustment for multiple comparisons
☐	☒	A full description of the statistical parameters including central tendency (e.g. means) or other basic estimates (e.g. regression coefficient) AND variation (e.g. standard deviation) or associated estimates of uncertainty (e.g. confidence intervals)
☒	☐	For null hypothesis testing, the test statistic (e.g. F, t, r) with confidence intervals, effect sizes, degrees of freedom and P value noted<br>*Give P values as exact values whenever suitable.*
☒	☐	For Bayesian analysis, information on the choice of priors and Markov chain Monte Carlo settings
☒	☐	For hierarchical and complex designs, identification of the appropriate level for tests and full reporting of outcomes
☒	☐	Estimates of effect sizes (e.g. Cohen's d, Pearson's r), indicating how they were calculated

Our web collection on statistics for biologists contains articles on many of the points above.

Software and code

Policy information about availability of computer code

Data collection	The luciferase activity was collected using Gen5 software.<br>The SPR data were collected using Pioneer FE software.<br>Cryo-EM data were acquired at the HHMI Janelia Research Campus on a Titan Krios transmission electron microscope (ThermoFisher) operating at 300 keV and equipped with a Gatan K3 Summit direct electron detector using SerialEM software at super-resolution mode.
Data analysis	The IFN luciferase reporter assay data were further processed with GraphPad Prism.<br>SPR data analysis was performed using Qdat software (Sartorius).<br>The cryo-EM dataset was processed through Relion and as described in the methods section of the manuscript.

For manuscripts utilizing custom algorithms or software that are central to the research but not yet described in published literature, software must be made available to editors and reviewers. We strongly encourage code deposition in a community repository (e.g. GitHub). See the Nature Portfolio guidelines for submitting code & software for further information.

Data

Policy information about availability of data

All manuscripts must include a data availability statement. This statement should provide the following information, where applicable:

- Accession codes, unique identifiers, or web links for publicly available datasets
- A description of any restrictions on data availability
- For clinical datasets or third party data, please ensure that the statement adheres to our policy

The atomic coordinates and cryo-EM maps generated in this study have been deposited in EMDB and PDB as follows: end-end RIG-I:p3dsRNA24:Riplet complex (EMDB: 29823 [https://www.ebi.ac.uk/emdb/EMD-29823], PDB: 8G7T [https://doi.org/10.2210/pdb8G7T/pdb]), end-semi-closed-end RIG-I:p3dsRNA24:Riplet complex (EMDB: 29824 [https://www.ebi.ac.uk/emdb/EMD-29824], PDB: 8G7U [https://doi.org/10.2210/pdb8G7U/pdb]) and end-inter RIG-I:p3dsRNA24:Riplet complex (EMDB: 29825 [https://www.ebi.ac.uk/emdb/EMD-29823], PDB: 8G7V [https://doi.org/10.2210/pdb8G7V/pdb]). The raw cryo-EM micrographs have been deposited in EMPIAR (EMPIAR-11494 [https://www.ebi.ac.uk/empiar/EMPIAR-11494/]). The raw micrographs of EMSA and immunoblotting have been provided as source data. The raw data of IFN reporter assay have been provided as source data. Source data are provided with this paper.

Research involving human participants, their data, or biological material

Policy information about studies with human participants or human data. See also policy information about sex, gender (identity/presentation), and sexual orientation and race, ethnicity and racism.

Reporting on sex and gender	N/A
Reporting on race, ethnicity, or other socially relevant groupings	N/A
Population characteristics	N/A
Recruitment	N/A
Ethics oversight	N/A

Note that full information on the approval of the study protocol must also be provided in the manuscript.

Field-specific reporting

Please select the one below that is the best fit for your research. If you are not sure, read the appropriate sections before making your selection.

[x] Life sciences [ ] Behavioural & social sciences [ ] Ecological, evolutionary & environmental sciences

For a reference copy of the document with all sections, see nature.com/documents/nr-reporting-summary-flat.pdf

Life sciences study design

All studies must disclose on these points even when the disclosure is negative.

Sample size	No statistical methods were used to predetermine sample sizes. Sample sizes were selected empirically from our previous experimental experience (PMID: 36272408; PMID: 30784585; PMID: 31851914; PMID: 28180316; PMID: 26371557).
Data exclusions	No data was excluded from our analyses.
Replication	Except for the cryo-EM studies (one time), SPR (one time) and WB (two times), each experiment was repeated three times at least. All repeated experiments reproduce the results.
Randomization	For cell experiments, all cells in each experiment were from the same pool of parental or knockout cells.
Blinding	The investigators were not blinded because each experiment was carried out by one person. However, the samples were added properly. The data analysis were strictly done with all data points included. Therefore, blinding is not possible in this study but does not affect the results.

Reporting for specific materials, systems and methods

We require information from authors about some types of materials, experimental systems and methods used in many studies. Here, indicate whether each material, system or method listed is relevant to your study. If you are not sure if a list item applies to your research, read the appropriate section before selecting a response.

Materials & experimental systems

n/a	Involved in the study
☐	☒ Antibodies
☐	☒ Eukaryotic cell lines
☒	☐ Palaeontology and archaeology
☒	☐ Animals and other organisms
☒	☐ Clinical data
☒	☐ Dual use research of concern
☒	☐ Plants

Methods

n/a	Involved in the study
☒	☐ ChIP-seq
☒	☐ Flow cytometry
☒	☐ MRI-based neuroimaging

Antibodies

Antibodies used

The antibodies used in this study target human proteins. Anti-Riplet, Sigma, HPA021576, polyclonal, 1:1000; Anti-GAPDH, Santa Cruz Biotech, sc-47724, monoclonal, 1:1000.

Validation

All primary antibodies from commercial vendors are validated by the manufacturers for the species and assay in our study. Validation data are available from the manufacturers' websites as follows:

immunoblotting
Anti-Riplet: Sigma, HPA021576, polyclonal, 1:1000
Species: Human; biological source: rabbit
Application: WB (1:1000)
https://www.sigmaaldrich.com/US/en/product/sigma/hpa021576

immunoblotting
Anti-GAPDH: Santa Cruz Biotech, sc-47724, monoclonal, 1:1000
Species: Human; biological source: mouse
Application: WB (1:100 to 1:1000), IP (1:50-1:500), IF (1:50-1:500), IHC (1:50-1:500)
https://www.scbt.com/p/gapdh-antibody-0411

Eukaryotic cell lines

Policy information about cell lines and Sex and Gender in Research

Cell line source(s)

The HEK293T cells derived from a female patient were purchased from ATCC. The HEK293T-Riplet-KO cells were generated in Pyle lab using CRISPR-Cas9 technique.

Authentication

The HEK293T cells from ATCC were not authenticated. The HEK293T-Riplet-KO cells were evaluated using genotyping and immunoblotting.

Mycoplasma contamination

The cell line was not tested for mycoplasma contamination.

Commonly misidentified lines
(See ICLAC register)

No commonly misidentified cell lines were used in this study.

Plants

Seed stocks

N/A

Novel plant genotypes

N/A

Authentication

N/A
